# Key methodological considerations for usability testing of electronic patient-reported outcome (ePRO) systems

**DOI:** 10.1007/s11136-019-02329-z

**Published:** 2019-10-18

**Authors:** Olalekan Lee Aiyegbusi

**Affiliations:** Centre for Patient Reported Outcome Research, Institute of Applied Health Research, University of Birmingham, Edgbaston, Birmingham, B15 2TT UK

**Keywords:** Usability testing, Electronic patient-reported outcomes, PROs, ePROs, ePRO systems, ePROM, Digital health, eHealth, Telehealth, Electronic systems

## Abstract

**Introduction:**

Recent advances in information technology and improved access to the internet have led to a rapid increase in the adoption and ownership of electronic devices such as touch screen smartphones and tablet computers. This has also led to a renewed interest in the field of digital health also referred to as telehealth or electronic health (eHealth). There is now a drive to collect these PROs electronically using ePRO systems.

**Method:**

However, the user interfaces of ePRO systems need to be adequately assessed to ensure they are not only fit for purpose but also acceptable to patients who are the end users. Usability testing is a technique that involves the testing of systems, products or websites with participants drawn from the target population. Usability testing can assist ePRO developers in the evaluation of ePRO user interface. The complexity of ePRO systems; stage of development; metrics to measure; and the use of scenarios, moderators and appropriate sample sizes are key methodological issues to consider when planning usability tests.

**Conclusion:**

The findings from usability testing may facilitate the improvement of ePRO systems making them more usable and acceptable to end users. This may in turn improve the adoption of ePRO systems post-implementation. This article highlights the key methodological issues to consider and address when planning usability testing of ePRO systems.

## Introduction

Recent advances in information technology and improved access to the internet have led to a rapid increase in the adoption and ownership of electronic devices such as touch screen smartphones and tablet computers. In 2017, about 77% of American adults reported owning a smartphone compared to 35% in 2011 [1]. The increase in ownership of electronic devices has also been observed worldwide albeit at a lower rate in developing countries [1] and the digital divide between younger and older populations has narrowed over the past decade [2].

These developments have in turn led to an upsurge of interest in digital healthcare also known as telehealth. It is now feasible to remotely collect patient-reported outcomes (PROs) using electronic devices. A PRO can be defined as “any report of the status of a patient’s health condition that comes directly from the patient, without interpretation of the patient’s response by a clinician or anyone else” [3]. An ePRO is therefore a PRO that is collected electronically. In the past, PROs were mainly collected using paper formats which were associated with significant administrative burden, missing data and data entry errors.

EPROs are increasingly used in clinical trials and cohort studies to appraise, from a patient perspective, the effectiveness and safety of interventions [4]. This is important as regulatory authorities are now paying greater attention to PRO data when making decisions about drug approvals [5–7]. The use of ePROs instead of paper formats in clinical trials could facilitate the robust analysis and reporting of PRO data which is often neglected or inadequate by making the data available in easily exportable formats with fewer errors and missing data [8].

Clinicians are now able to use interactive electronic patient-reported outcome (ePRO) systems to monitor and deliver healthcare to a considerable number of patients. Patients can access ePRO systems using mobile devices to provide feedback on their health status and response to treatments in ‘real time’ [9, 10]. The use of telehealth could therefore facilitate patient engagement with care which is a key element of delivering patient-centred care. It has also been demonstrated that patient reports of their health could complement clinical and laboratory parameters in routine clinical practice [11, 12]. Recent research suggest that the use of ePRO systems could facilitate the remote monitoring of patients [13]; enhance efficiency by reducing the need for hospital appointments [14]; and improve patient outcomes such as quality of life and survival rates [15]. The number of health care providers developing ePRO systems has increased in recent years [16, 17] and is set to rise considerably in future as more evidence to support their use become available.

It is therefore crucial that the user-friendliness and usability of the ePRO user interfaces are adequately assessed and improved throughout system development to reduce attrition rates in clinical trials and enhance their adoption post-implementation in clinical practice.

This article highlights the important issues that need to be considered and addressed when planning the usability testing of ePRO systems. Although ePRO systems are the primary focus, majority of the issues discussed are relevant for usability testing of websites or other types of systems that involve human–computer interaction. This paper is focused on methodological considerations for planning usability tests in the context of ePRO systems rather than the design of user interface. However, guidance and recommendations for the design of user interfaces are available in various publications and guidelines [18–23].

## Usability and usability testing

According to the International Organization for Standardization (ISO), usability is an outcome of use which can be defined as “the extent to which a system, product or service can be used by specified users to achieve specified goals with effectiveness, efficiency and satisfaction in a specified context of use” [23].

Therefore, usability testing can be described as the formal assessment of the extent to which interaction with a product or system is effective, efficient and perceived as satisfactory by users. It allows end users to actually test ePRO systems and provides developers the opportunity to evaluate usability.

Based on the ISO definition, the three measures of usability are effectiveness, efficiency and satisfaction. Effectiveness refers to the ability of participants to perform tasks in order to achieve pre-determined goals completely and accurately without negative consequences [23, 24]. A negative consequence in the case of an ePRO system might be the accidental selection of a questionnaire option (due to suboptimal interface layout) which would send a red alert to clinicians. Efficiency relates to the amount of resources required by participants to achieve the pre-specified goals. An effective and efficient system or product could be considered as one that offers a better way of achieving specific goals compared to the current manner [25]. Satisfaction refers to the subjective opinions of participants based on their experience interacting with a system or product [23]. Some authors consider satisfaction with a system or product as equivalent to desirability the presence of which might actually facilitate the adoption of a system or product with flawed effectiveness and efficiency [25]. However, it can be argued that a system with effectiveness or efficiency issues would soon be abandoned regardless of initial desirability. An ePRO system needs to be rated highly on the three measures of usability to be considered fit for purpose. In turn, a system perceived as fit for purpose has a better chance of adoption [26] by patients and clinicians post-implementation.

The context of use refers to the characteristics of the users, tasks, equipment and the physical and social setting in which a system or product is used [23].

Evaluation of the measures of usability may be achieved by recruiting participants from the target population to perform pre-determined tasks using the product or system and provide feedback on their user experiences. Usability testing also provides ePRO developers the opportunity to detect and fix issues early during system development. It is important that usability testing is conducted iteratively [27] during system development to ensure that issues are detected and addressed adequately prior to full-scale implementation. This ensures that the final product or system is fit for purpose and may reduce attrition rates post-implementation [28].

## Key points to consider when planning a usability test

### Complexity of the user pathway and the ePRO system

An ePRO system is typically nestled within a broader IT system and usually requires users to perform a number of tasks before the ePRO questionnaires can be accessed. These may include tasks such as navigating webpages by following url links, entering personal details for verification before gaining access to the ePRO portal and login out of the system. Usability testing should assess the entire pathway and identify potential issues as its user-friendliness is crucial for user adoption. The complexity of an ePRO system also needs to be assessed when planning its usability testing. Most ePRO systems involve the adaptation of existing paper PRO questionnaires [24, 29]. A basic adaptation keeps the electronic version as identical to the paper version as possible. It involves minor modifications to format or questions and such systems usually have a low level of complexity. Moderate adaptations may include subtle changes to meanings and format such as text font, colour or size. An extensive adaptation entails substantial changes such as the removal of items or the addition of functions such as drop down menus leading to the development of a more complex or sophisticated system [19, 30]. The greater the modification and in turn the complexity of an ePRO system, the larger the overall sample size and the number of test cycles that might be required [31]. In addition, the greater the degree of modification of an existing paper questionnaire, the greater the likelihood that additional studies such as psychometric validation might be required to evaluate the electronic version [19, 30].

### Stage of system development

**Fig. 1:**
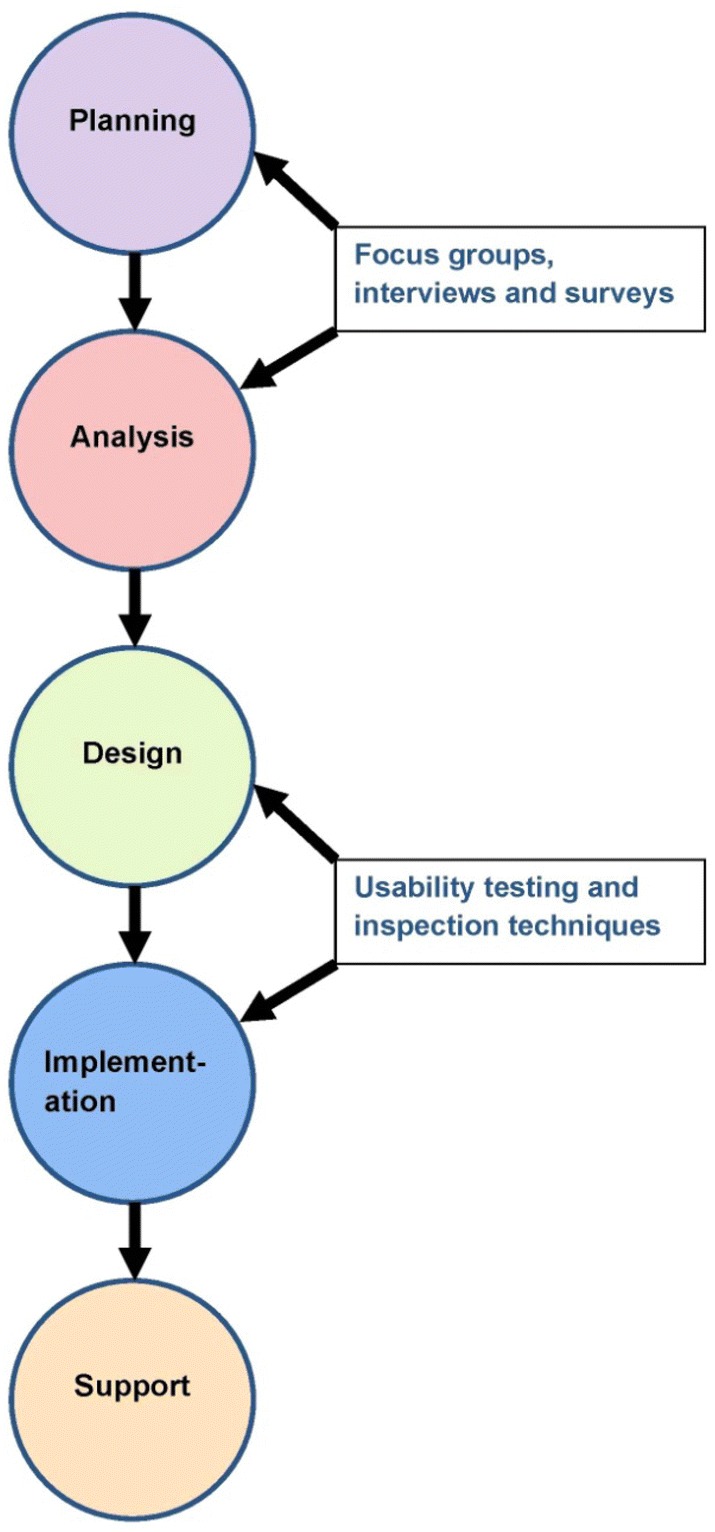
Relationship between the stages of system development and applicable methods

While the focus of this article is usability testing, it is worth mentioning that it is one of a number of study methods utilised during system development. System development can be divided into five stages, namely (i) planning, (ii) analysis, (iii) design, (iv) implementation and (iv) support [32]. Various study methods can be utilised during these stages to ensure that user requirements are met and the ePRO system is therefore fit for purpose. For instance, interviews, focus groups and surveys may be conducted with stakeholders during the planning and analysis stages, while usability testing and inspection techniques such as heuristic evaluation and cognitive walkthroughs may be conducted during design and implementation stages [32]. Figure 1 depicts the relationship between the stages of system development and the methods applicable.

It is important to consider the stage of system development as this would determine the type and depth of usability testing to conduct. Broadly speaking, there are two types of usability testing: formative and summative testing [33]. Formative testing is usually performed during the early stages of system development and the aim is to identify major (usually technical) issues. Formative usability testing may be conducted before any development work is done during the design phase using wireframes (mock-up screens) [34]. Formative test sessions are often less formal with greater interaction between participants and moderator. Formative tests collect mainly qualitative data especially during the earlier cycles, although some quantitative data such as error rate may also be collected [35]. After a series of iterative formative testing, the number of which might depend on the amount of issues detected by participants interacting with the system, summative testing may be conducted. The aim of summative testing is to obtain definitive evidence of usability [25] which may be used to support government regulatory claims or marketing campaigns. Summative test sessions are usually more formal with little or no interaction with a moderator.

Summative tests usually involve closer observation and recording of participants’ actions as well as the collection of more quantitative data such as success or failure on tasks, average time on task, completion rates and error rates for statistical calculations [25]. As summative testing involves more statistical analyses, it requires more participants than formative testing. The issue of sample sizes is discussed further in the dedicated section.

The stage of ePRO development would also determine whether tests are conducted on-site or off-site (often at participants’ homes). During the early stages, on-site moderated tests are more appropriate as these provide the opportunity to observe how well participants interact with a system. However, later on testing should be done remotely within participants’ own environment. Remote testing may be synchronous or asynchronous. In synchronous, the session is facilitated and data are collected by the evaluators in real time, while in asynchronous the session is not facilitated and the evaluator only has access to the data after the session has ended [36]. As off-site testing more closely resembles real life use, a successful test may provide ePRO developers the assurance that a system is indeed usable. However, a number of studies have demonstrated that remote synchronous usability tests may provide comparable results to traditional on-site tests of the same website or system, while participants of asynchronous tests may require more time to complete tasks [37, 38]. Remote testing may help developers detect potential internet, software or hardware compatibility issues.

### Usability metrics to measure

Usability metrics to measure may be grouped into three categories: self-reported, observer-reported and implicit [39]. Self-reported metrics come directly from participants and include satisfaction and difficulty ratings. Observer-reported relates to assessments of participants’ actions by the evaluator. Observer-reported metrics include time to complete tasks. Self- and observer-reported metrics may suffer from bias as participants often consider their responses and are conscious of their actions and may not act as they would in real life [40, 41]. Implicit metrics which are less commonly used may provide the most unbiased data as they measure participants’ unconscious behaviours and physiology [35]. These include eye tracking and pupillary dilation [42].

Usability metrics relevant to ePRO systems are linked to the measures of usability (i.e. effectiveness, efficiency and satisfaction). Relevant quantitative metrics for effectiveness include error rates and completion rates. Time required for completing tasks, numbers of clicks to complete tasks, and cost effectiveness are appropriate metrics for efficiency. Overall satisfaction rates and proportion of users reporting complaints can be used to assess satisfaction [35]. While effectiveness, efficiency and satisfaction are often assessed quantitatively, they could also be assessed qualitatively. For example, effectiveness could be assessed by discussing errors and successful task completions with participants. Participants could also describe their satisfaction with the system in their own words [35].

The choice and number of metrics to measure may be influenced by the type of usability testing being conducted. As mentioned earlier, formative testing may involve the measurement of fewer quantitative metrics; relying more on qualitative feedback from participants while summative testing, which often involves more statistical analyses, tends to require the measurement of more quantitative metrics.

### The use of usability questionnaires

Developers of ePRO systems could use usability questionnaires to capture and quantify participants’ subjective opinion and satisfaction with their ePRO interfaces. Some questionnaires are designed for specific interfaces such as the Website Analysis and Measurement Inventory for websites [43]. Others, such as the System Usability Scale (SUS) [44], are more generic and can be used across interfaces. The use of such scales provides the opportunity to generate additional data which could be analysed to generate useful statistics about ePRO systems. Participants’ scores from each test cycle may be compared with previous scores to confirm any improvements in satisfaction with the system. However, not all developers perceive usability scales as pertinent and some studies have suggested that a qualitative approach might be more useful especially in studies involving older participants [17, 45]. Once again it is vital that the goals of the developers and stakeholders are considered when making decisions about using usability scales.

### Sample size

There has been considerable debate about the appropriate sample size for usability testing [31, 46–50]. Testing with more participants than necessary would increase costs and project time [51]. On the other hand, important issues might go undetected if inadequate sample sizes are used. In reality, there are no magic formulas for calculating sample sizes. The decision needs to be based on the careful consideration of a number of factors, namely (i) iterative nature of usability testing, (ii) homogeneity of target end users, (iii) complexity of the system and (iv) type of usability testing.

#### Iterative nature of usability testing

**Fig. 2:**
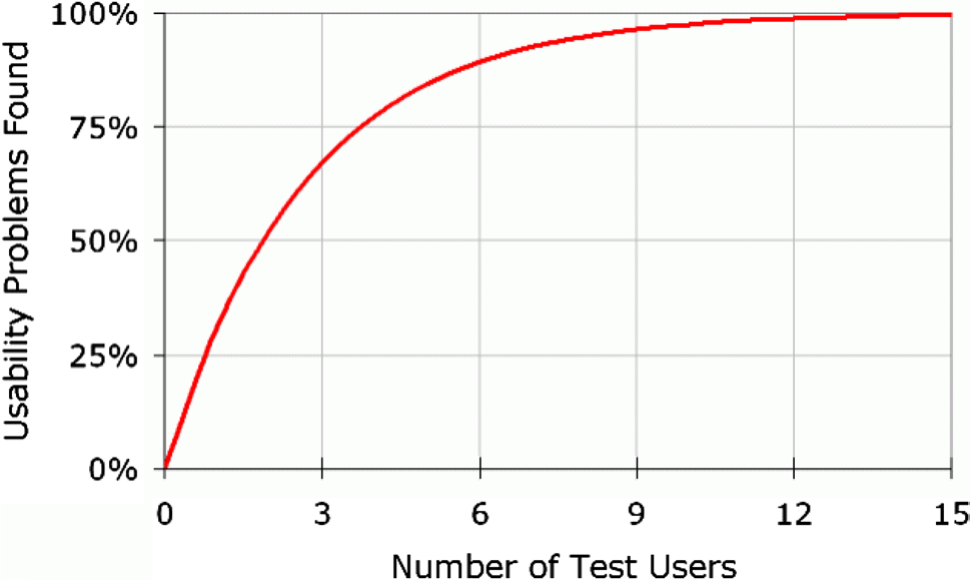
Sample size for usability cycles. Reproduced with the kind permission of the Nielsen Norman Group [53]

Studies have shown that five participants are required per (formative) test cycle to detect over 80% of issues (Fig. 2) [27, 52, 53]. However, as Spool and Schroeder demonstrated in their study, up to 15 participants might be required before serious usability issues are found [31]. Many system developers, usability personnel and researchers struggle to accept the recommendation of five users per test cycle as they are more familiar with larger sample requirements for most qualitative and quantitative studies. However, improving the usability of any system should be an iterative process which would allow developers the opportunity to detect and correct issues after each test cycle [27, 54]. It is therefore more sensible, for instance, to test with five participants per cycle and have the opportunity to detect issues and improve a system over four test cycles than to conduct a single cycle with 20 participants with no way of telling if subsequent changes to the system has improved its usability. It should be noted that this estimate of five participants per test cycle does not take into account the other three factors.

While the general expectation with iterative testing is that fewer issues will be detected with each test cycle until no substantial benefit is gained from further testing [54], it is quite possible that changes made on the basis of the results obtained from a cycle might inadvertently introduce fresh issues. Therefore, each cycle checks and assesses the changes made to the system. A ‘stopping rule’ needs to be agreed between the development team and commissioning body at the start of the project to prevent interminable testing [33]. An option is to stop further testing once the test results from a summative test meet pre-determined targets [33].

#### Homogeneity of target end users

The estimate of five participants per iteration is only appropriate if the target end users are reasonably homogenous in their socio-demographic characteristics. For instance, studies have shown that the age of participants might have a significant influence on usability experiences [21, 55]. Therefore, it is very likely that younger and older end users would have different satisfaction levels if they interact with the same system [56]. For this reason, if a system is being designed for use by both age groups, each should be treated as a distinct group when estimating sample sizes. There is a suggestion that fewer participants may be recruited per group per cycle as some overlap in participant experience is bound to occur [53]. However, decisions about sample sizes may also be dependent on the complexity of the system.

#### Design of ePRO systems

The International Society for Pharmacoeconomics and Outcomes Research (ISPOR) suggests that the complexity of the physical and cognitive tasks to be performed during a usability test may inform decisions about sample sizes [19]. The complexity of the tasks is influenced by the characteristics of the PRO questionnaire. Questionnaires that utilise matrices and drop down options are more complex compared to those with simple line by line formats. The current recommendation is 5 to 10 participants for simple ePRO systems and up to 20 for more physically and cognitively demanding systems [19]. However, these ranges are fairly wide and it is not clear whether the recommendation refers to each test cycle or the entire test [19].

How the system would be accessed may also influence sample size requirements as different platforms may have different usability issues [56]. The rapid developments in mobile technology have led to an increasing number of people accessing websites and web applications using mobile devices such as smartphones, tablets and phablets rather than ‘traditional’ desktops and laptops. Producing a version of an ePRO system for each type of device is probably impossible given the vast number of variations in screen sizes and resolutions. Responsive web design (RWD) an approach that allows dynamic adaptations to various screen sizes, resolutions and orientations is regarded as a solution [57]. Usability testing of websites or ePRO systems designed using RWD should be done across multiple platforms [58]. However, it is impractical to conduct usability testing for all types of devices. Therefore, developers have to decide which key platforms to test based on the degrees of similarity or differences between groups of devices. The recommendation of five subjects per test cycle should be applied to each device type (i.e. five subjects per device type per cycle) as user experiences may completely differ from one view of the ePRO system to another [58].

Touch screen devices such as smartphones and tablets may be easier to use and control than desktops or laptops which require keyboards and mouse. However, they usually have smaller screens which might influence the visual display and font sizes of ePROs. This could be an issue for participants with poor eyesight and this should be considered when selecting study participants [24]. The United States Access Board and the World Wide Web Consortium have published detailed guidelines to improve IT accessibility for all individuals regardless of disability [59–61].

#### Type of usability testing

Sample size requirements would also be influenced by the type of testing being conducted which would in turn be determined by the objectives of the developers. As the data collected during formative usability testing (especially during the early stages) tends to be more qualitative than quantitative, sample size will be influenced by the theoretical approach and the achievement of thematic or data saturation [62, 63]. Summative usability testing would need more participants to ensure that statistical tests are adequately powered and the results meaningful [49]. Sample size calculations for controlled experiments would depend on study design, estimates of the variance and the desired level of precision (which includes the size of the critical difference and the chosen confidence level) [51, 64]. However, detailed discussion of sample size calculations for statistical tests is outside the scope of this present article.

### Task scenarios

The setting for usability testing is by nature artificial and moderator-controlled. Despite this situation, participants are expected to interact with a system, website or product as they would ‘normally’ do without observation or guidance. It should be expected that in reality people will often behave differently during moderated on-site test sessions and un-moderated off-site testing. It is therefore necessary for the moderator to set the scene by providing suitable scenarios which give context and meaning to the tasks to be performed in order to achieve pre-determined goals. Scenarios should ideally mirror the types of outcome that may be obtained in real life. The platforms participants use for their tests will determine their pathway to accessing the ePRO system and in turn the applicable scenarios. For example, participants using a laptop might have to carry out some initial navigation by following web links, whereas smartphone users might only have to tap the icon for the ePRO application on their phones. The number of scenarios to use for a particular test session will depend on the number of possible outcomes for interaction with the ePRO system. This is especially relevant for ePRO systems that employ conditional branching (skip logic) where the sequence of questions is determined by participants’ responses [65]. As there are a higher number of possible paths patients may take, there may be a need for more scenarios especially as not all questions may be formatted the same way. For instance, an item on ‘pain’ might have an initial ‘yes or no’ option. Individuals who click ‘no’ would move on to the next symptom, whereas those who select ‘yes’ would have a further item such as a visual analogue scale (VAS) ruler appear. Therefore, participant A’s interaction and experience with the system may not be the same as that of participant B. Crafting realistic scenarios requires skill and a delicate balance has to be achieved with information provision. Participants should be given just enough information to execute the tasks [66].

### Moderator’s duties and choice of moderating technique(s)

Usability test sessions, particularly formative ones, need to be effectively moderated in order to derive useful insights which can subsequently be used for system improvement. Moderating usability tests is a skilled task that requires excellent judgement and observational skills. The degree of interaction between the moderator and the participants should be decided prior to the start of the testing cycle. As discussed earlier this would generally be determined by the type of usability testing to be conducted. The moderator needs to clarify before each test session that the purpose of the session is to evaluate the *interface* of the system and not to assess the *meaning and relevance* of the individual questions of the ePRO questionnaire. It is important that the moderator understands and makes this distinction as participants may confuse the two activities. For instance, they may comment on the clarity or suitability of individual questions rather than the font size of the interface. Content validation to evaluate the meaning and relevance of questions should be separately conducted for newly developed or extensively modified existing questionnaires.

There are four moderating techniques described in the literature [67], namely (i) concurrent think aloud (CTA), (ii) retrospective think aloud (RTA), (iii) concurrent probing (CP) and (iv) retrospective probing (RP).

In CTA, participants are encouraged to ‘think aloud’ and vocalise their thoughts on the user interface as they interact with the system or website and execute the pre-determined tasks. The moderator employs minimal prompts to keep participants talking. With RTA, the test sessions are usually video recorded and participants complete their tests in silence. The moderator then asks them afterwards to recall and vocalise their thoughts during the test usually with the aid of the video recording [68]. The only technique in which the moderator plays an active role during test sessions is CP. In CP, the moderator asks probing or follow-up questions to participants’ comments, non-verbal cues or noteworthy actions. When using RP, participants are allowed to complete their tests before being questioned by the moderator.

Each technique has its own advantages and disadvantages therefore the choice of technique to employ would depend on which qualities are important to system developers and stakeholders. Table 1 summarises these advantages and disadvantages [67]. A number of studies have compared moderating techniques [68]. It has been suggested that both ‘think aloud’ and retrospective approaches produce similar results which are prone to positive bias [18]. Participants in CTA sessions may take more time and complete fewer tasks compared to those recruited for sessions moderated by retrospective methods [18]. An option is to employ more than one technique and RP is particularly suitable for combining with any of the others.

**Table 1:** Pros and cons of moderating techniques

Moderating techniques	Pros	Cons
Concurrent think aloud (CTA)	Understand participants’ thoughts as they occur and as they attempt to work through issues they encounter Elicit real-time feedback and emotional responses	Can interfere with usability metrics, such as accuracy and time on task
Retrospective think aloud (RTA)	Does not interfere with usability metrics	Overall session length increases Difficulty in remembering thoughts from up to an hour before = poor data
Concurrent probing (CP)	Understand participants’ thoughts as they attempt to work through a task	Interferes with natural thought process and progression that participants would make on their own, if uninterrupted
Retrospective probing (RP)	Does not interfere with usability metrics	Difficulty in remembering = poor data

Reproduced with the kind permission of Dr Jennifer Romano Bergstrom [67]

It is important to note that aside from moderating technique, moderator skills may have a significant impact on the conduct and outcome of tests. For instance, RP relies heavily on the moderator’s ability to observe and note participants’ actions, verbal and non-verbal cues during the tests for subsequent probing.

## Conclusions

As advances in information technology continue and the adoption of mobile devices increases, digital healthcare will play a more prominent role in patient care. The development and use of ePRO systems could enhance the quality of clinical trials, and facilitate the remote monitoring and timely delivery of healthcare to patients. They could also promote patient involvement which is a crucial element of patient-centred care. However, the usability of the user interface of these systems needs to be adequately assessed by individuals drawn from the target population. The insights obtained from usability tests may be used to optimise ePRO systems to ensure that they are fit for purpose and acceptable to the end users.
